# Supplementary material for: Collagen prolyl 4-hydroxylase 1 is essential for HIF-1α stabilization and TNBC chemoresistance
Source: Nat Commun. 2018 Oct 26;9:4456. doi: 10.1038/s41467-018-06893-9 (PMC6203834; doi:10.1038/s41467-018-06893-9)
Supplement: Supplementary file 1 — Supplementary Information [file 41467_2018_6893_MOESM1_ESM.docx]

**Supplementary Information**

**Collagen prolyl 4-hydroxylase 1 is essential for**

**HIF-1α stabilization and TNBC chemoresistance**

**Xiong et al.**

**Supplementary Figures**

**
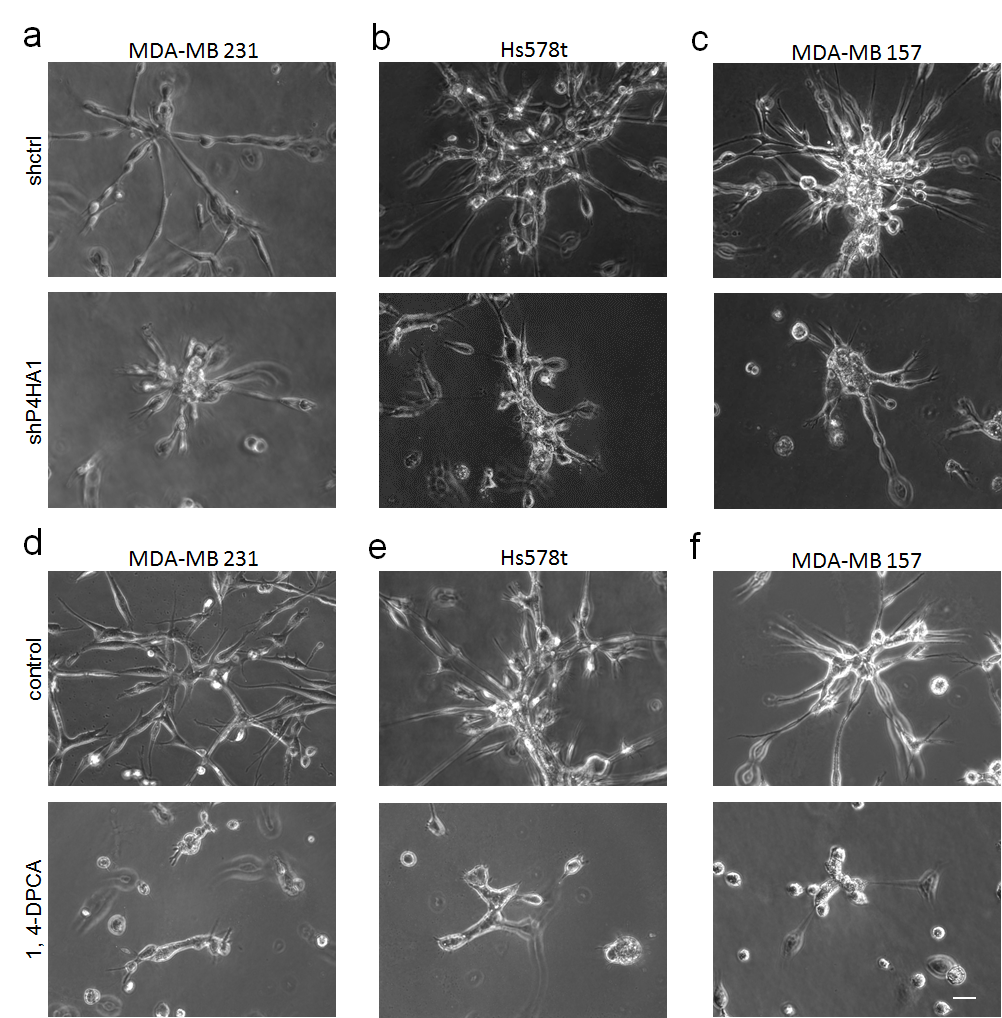
**

**Supplementary Figure 1. Control and P4HA1-silenced TNBC cell lines phenotypes in three-dimensional (3D) culture. a-c** Represented phase images of shctrl and shP4HA1 MDA-MB-231 cells, Hs578t, MDA-MB-157 cells in 3D culture. **d-f** Represented phase images of MDA-MB-231 cells, Hs578T, MDA-MB-157 cells treated with 1,4-DPCA, a P4H inhibitor, in 3D culture. Bar, 40 µm.


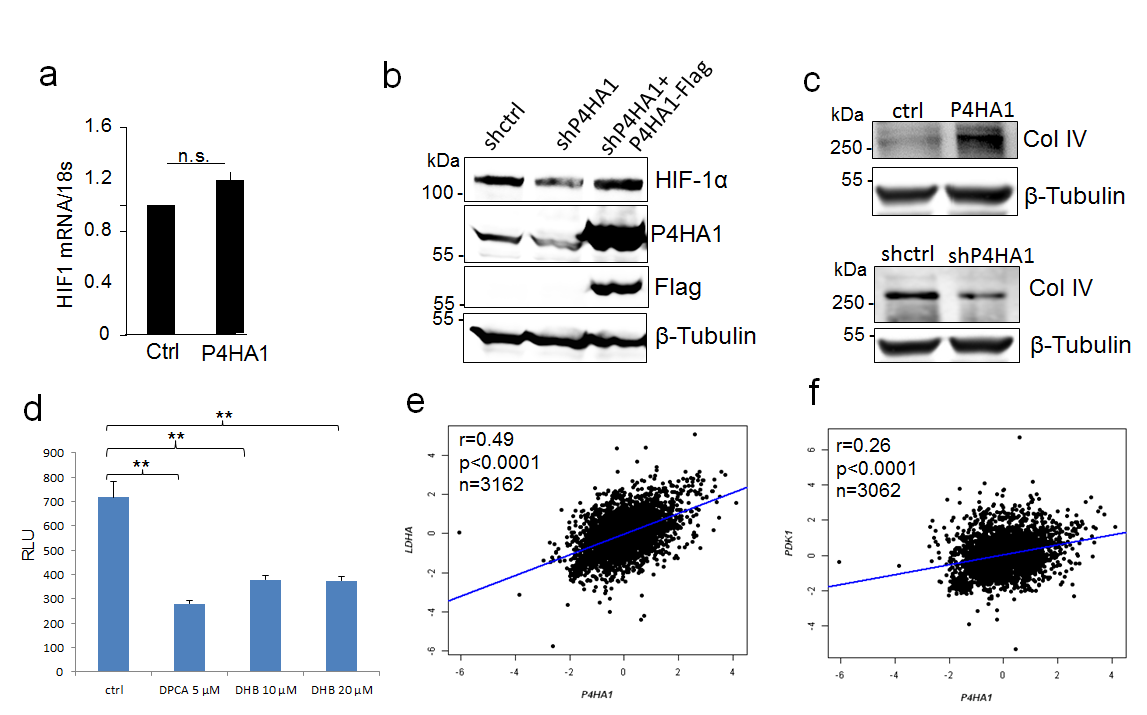


**Supplementary Figure 2. P4HA1 regulates transcriptional activation of HIF-1.** **a** Quantitative RT-PCR analyses of HIF-1α expression in control and P4HA1-expressing MCF10A lines; n.s., not significant. **b** HIF-1α and P4HA1protein levels were examined by western blot in control, P4HA1 silenced MDA-MB-231 cells, and P4HA1-silenced MDA-MB-231 cells with reconstituted P4HA1 expression. **c** Secreted collagen IV protein levels in control and P4HA1 expression MCF10A cells, control and P4HA1-silenced MDA-MB-231 cells were examined by western blot. **d** Luciferase activity was assessed in pGL-3HRE transfected 293FT cells after treatment with P4HA1 inhibitor 1,4-DPCA and DHB (n=3). Results are presented as mean ± SEM. **, p < 0.01, one-way ANOVA test. **e, f** The association between P4HA1 and expression and mRNA levels of LDHA and PDK1 in human breast cancer tissue. The data were analyzed by Pearson correlation analysis.


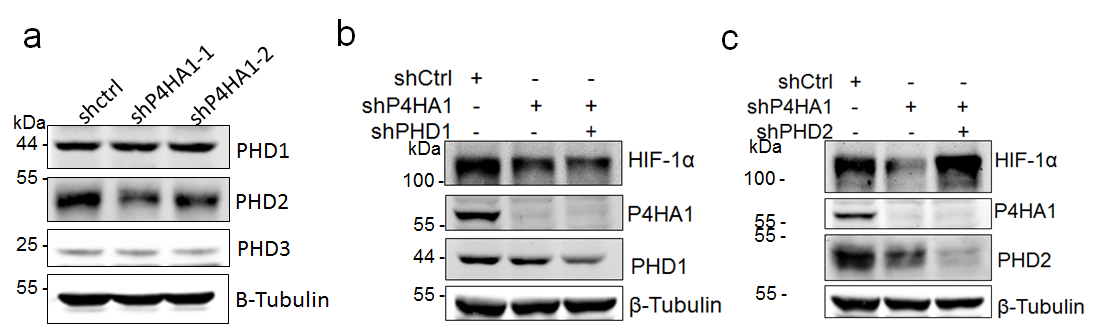


**Supplementary Figure 3. Roles of prolyl hydroxylases (PHD1, PHD2 and PHD3) in P4HA1-regulated HIF-1 stability. a** PHD1, PHD2 and PHD3 protein levels were assessed by western blot in control, P4HA1-silenced MDA-MB-231 cells. **b** HIF-1α protein level was examined by western blot in control, P4HA1-silenced MDA-MB-231 cells, and P4HA1, PHD1-double silenced MDA-MB-231 cells. **c** HIF-1α protein level was examined by western blot in control, P4HA1-silenced MDA-MB-231 cells, and P4HA1, PHD2-double silenced MDA-MB-231 cells.

**
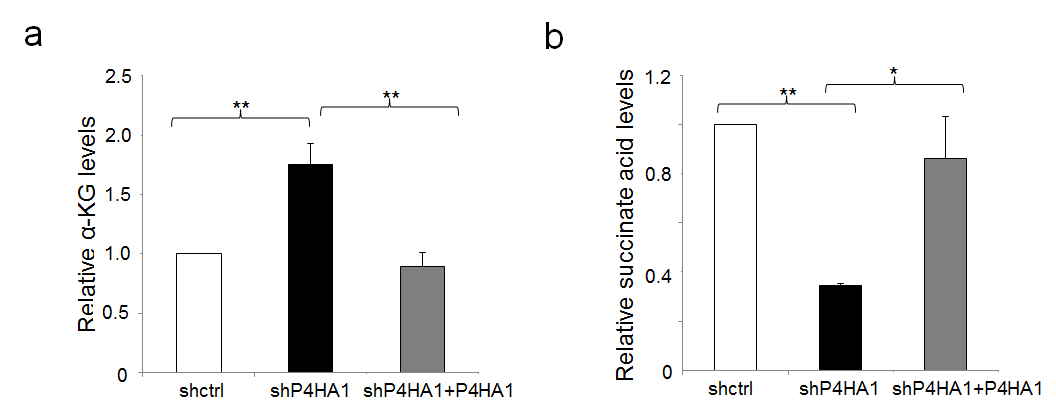
**

**Supplementary Figure 4. P4HA1 regulates α-KG levels and succinate levels. a** Total α-KG levels were measured in control, P4HA1-silenced MDA-MB-231, and P4HA1 silenced MDA-MB-231 cells with reconstituted P4HA1 expression. **b** Succinate levels were measured in control, P4HA1-silenced MDA-MB-231, and P4HA1 silenced MDA-MB-231 cells with reconstituted P4HA1 expression. n=3, results are presented as mean ± SEM. *, p < 0.05; **, p < 0.01, one-way ANOVA test.

**
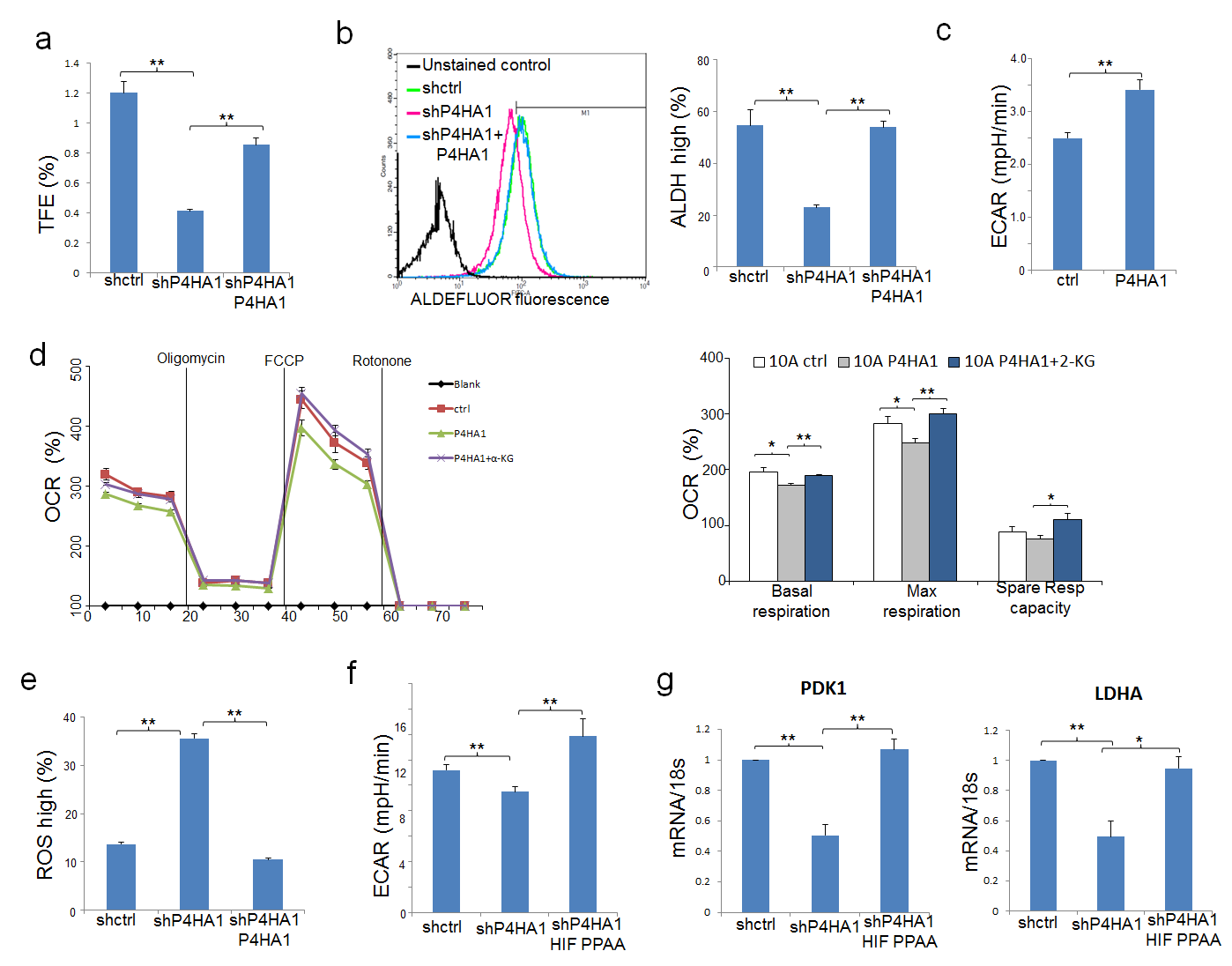
 Supplementary Figure 5. P4HA regulates oxidative phosphorylation in mammary epithelial cells. a** Quantification of tumorsphere formation efficiency (TFE) in control, P4HA1-silenced MDA-MB-231 cells, and P4HA1-silenced MDA-MB-231 cells with reconstituted P4HA1expression; n=6, results are presented as mean ± SEM. *, p < 0.05; **, p < 0.01, one-way ANOVA test. **b** FACS analysis of aldehyde dehydrogenase (ALDH) activity in control, P4HA1-silenced MDA-MB-231 cells, and P4HA1-silenced MDA-MB-231 cells with reconstituted P4HA1expression. Results are presented as mean ± SEM; n=3; **, p < 0.01, one-way ANOVA test. **c** Extracellular Acidification Rate (ECAR) was measured in control and P4HA1-expreession MCF10A cells; n=10, results are presented as mean ± SEM. **, p < 0.01, student’s t test. **d** Oxygen consumption rate (OCR) was assessed in control and P4HA1-expression MCF10A cells in the presence or absence of octyl-α-ketoglutarate; n=10, results are presented as mean ± SEM. *, p < 0.05, **, p < 0.01, one-way ANOVA test. **e** FACS quantification of ROS levels in control, P4HA1-silenced MDA-MB-231 cells, and P4HA1-silenced MDA-MB-231 cells with reconstituted P4HA1expression; n=3, results are presented as mean ± SEM; **, p < 0.01, one-way ANOVA test. **f** ECAR was measured in control, P4HA1-silenced MDA-MB 231 cells in the presence or absence of HIF-PPAA; n=10, results are presented as mean ± SEM. **, p < 0.01, one-way ANOVA test. **g** Quantification of mRNA levels of LDHA and PDK1 in control or P4HA1-silenced MDA-MB 231 cells in the presence or absence of HIF-PPAA; n=3, results are presented as mean ± SEM. *, p < 0.05; **, p < 0.01, one-way ANOVA test.


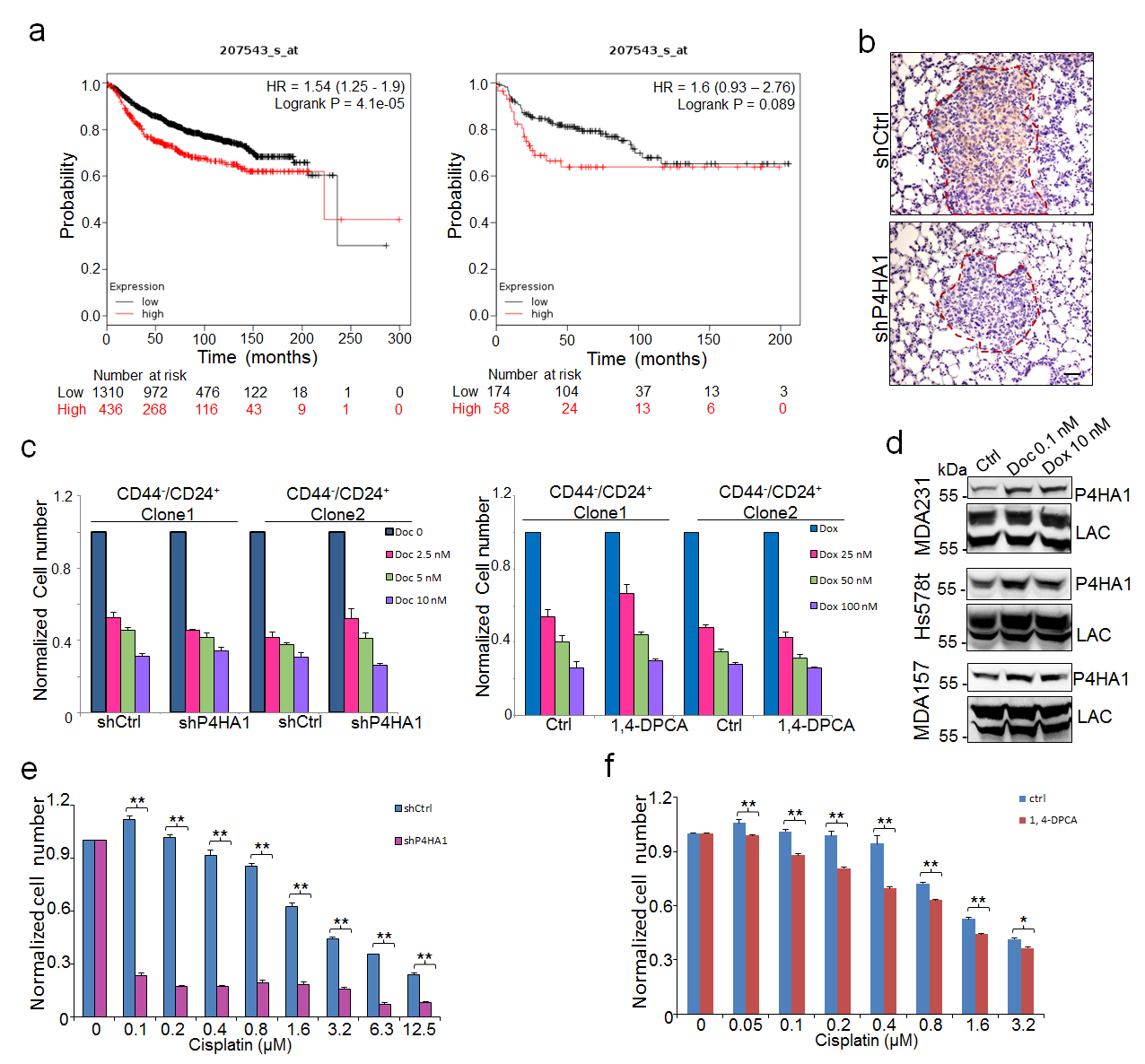


**Supplementary Figure 6. Roles of P4HA in chemoresistance of TNBC cell lines.**

**a** The association of P4HA1 expression in primary tumors with cancer metastasis was analyzed by Kaplan-Meier survival analysis in the published microarray dataset (http://kmplot.com). High P4HA1 mRNA levels (upper quartile) in breast cancer correlated with short distant metastasis free survival; p=4.1e-5, n=1746 (left panel, breast cancer); p=0.089, n=232 (right panel, basal-like), Kaplan-Meier survival analysis. **b** Represented HIF-1α IHC images of metastases colonization of control and P4HA1-silenced MDA-MB-231 cells in lung. Bar: 40 µm. **c** Quantification of cell number in control and shP4HA1 CD44^low^/CD24^+^ clone 1 and clone 2 after Docetaxel treatment (left); quantification of cell number after Doxorubicin treatment in the presence or absence of P4HA inhibitor (1,4-DPCA) (right). Results are presented as mean ± SEM; n=3. **d** MDA-MB-231, Hs578t, and MDA-MB 157 cells were treated with docetaxel (0.1 nM) and doxorubicin (10 nM) for 7 days, and P4HA1 expression levels were analyzed by western blot. **e** Quantification of cell number in control and shP4HA1 MDA-MB-231 cells after cisplatin treatment. Results are presented as mean ± SEM; n=3; **, p < 0.01, student’s t test. **f** Quantification of cell number in MDA-MB-231 cells after cisplatin treatment in the presence or absence of P4HA inhibitor (1,4-DPCA). Results are presented as mean ± SEM; n=4; *, p < 0.05; **, p < 0.01, student’s t test.

**
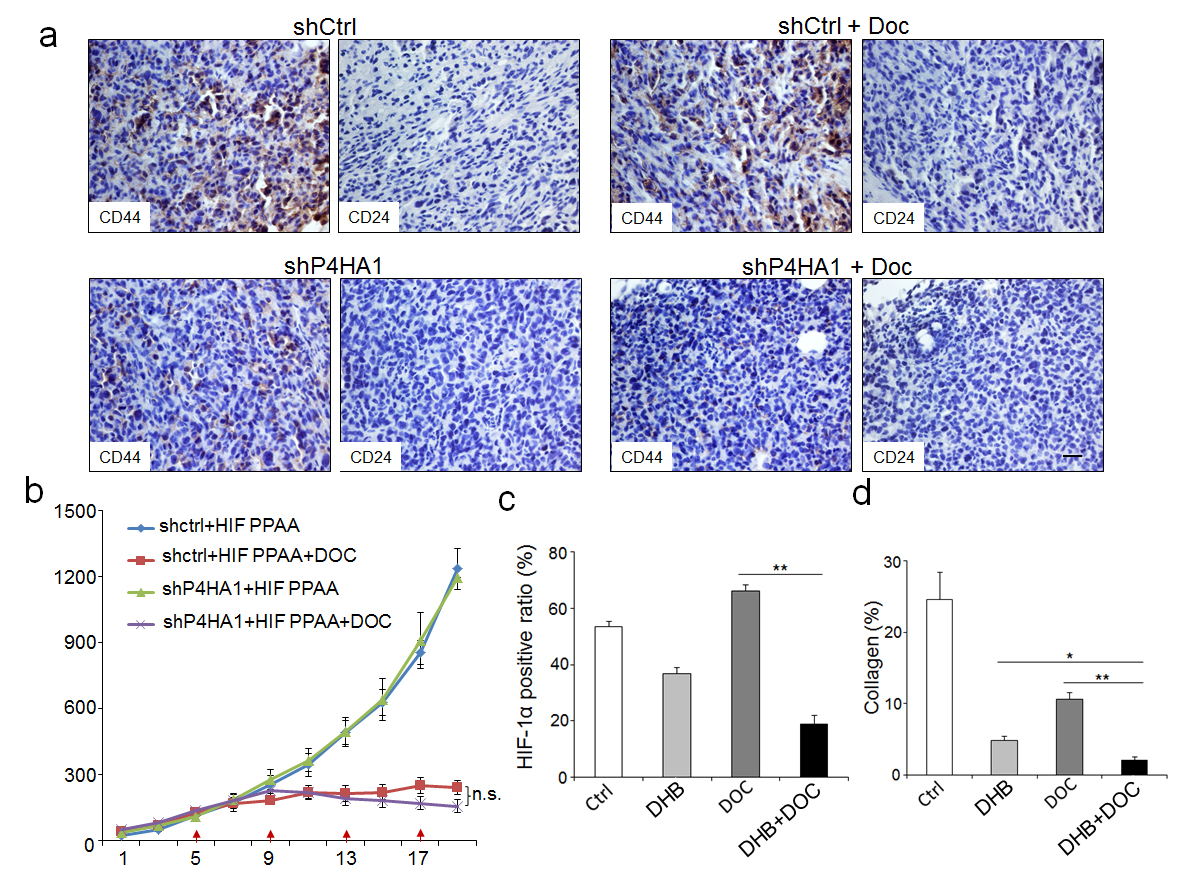
**

**Supplementary Figure 7. Inhibition of P4HA1 sensitizes TNBC to chemotherapeutic agent. a** Images of IHC staining (CD24 and CD44) in tumor sections from control and P4HA1-silenced MDA-MB-231 cells implanted mice treated with docetaxel. Bar: 20 µm. **b** Control and P4HA1-silenced HIF-PPAA expressing MDA-MB-231 cells were implanted into mouse fat pads, and mice were treated with docetaxel. Tumor growth curve was monitor during the treatment. Results are presented as mean ± SEM; n=9; n.s., not significant, one-way ANOVA test. **c, d** Quantification of HIF-1α levels and collagen levels in tumor sections from mice treated with DHB and/or docetaxel. Results are presented as mean ± SEM. n=4; *, p < 0.05; **, p < 0.01, one-way ANOVA test.

**
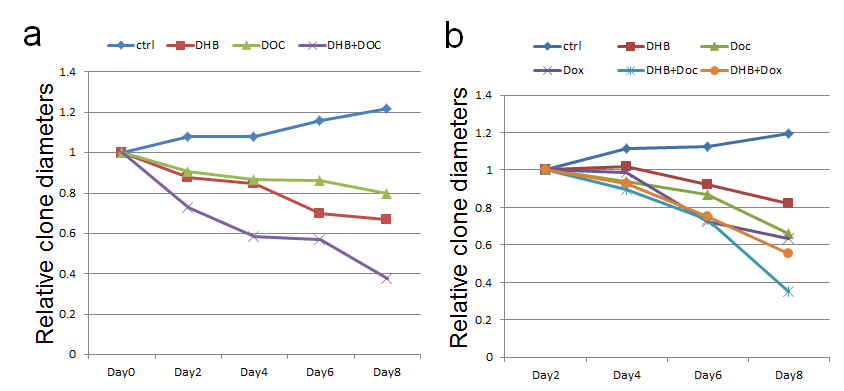
**

**
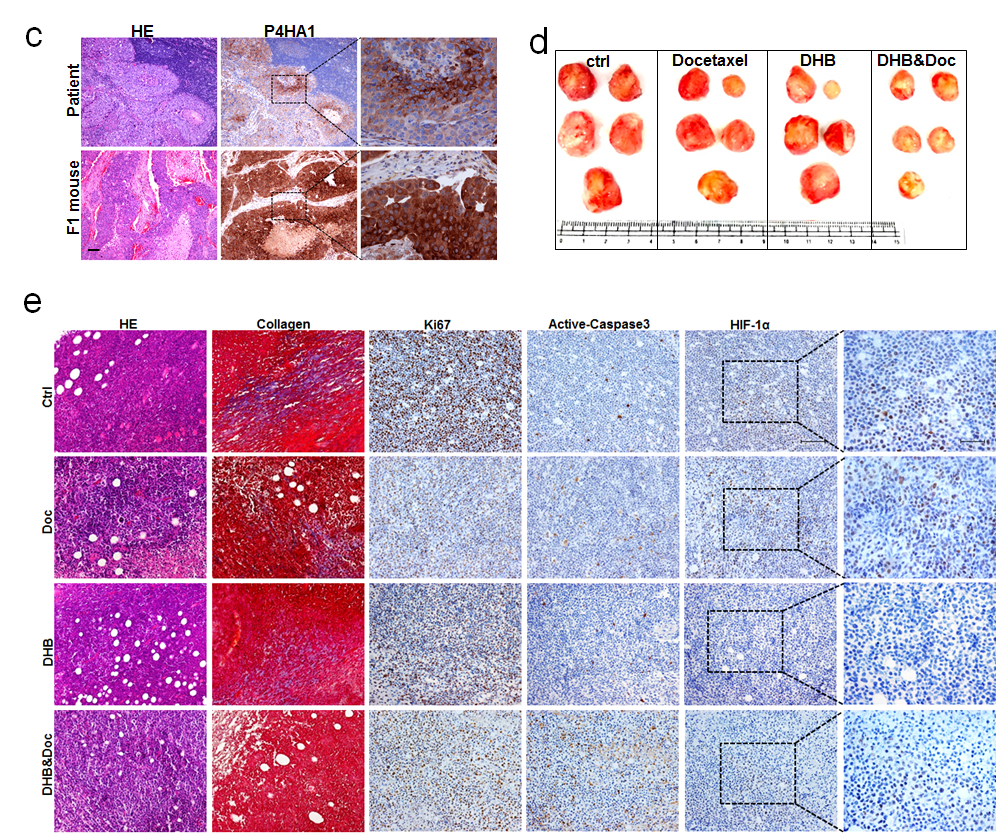
**

**
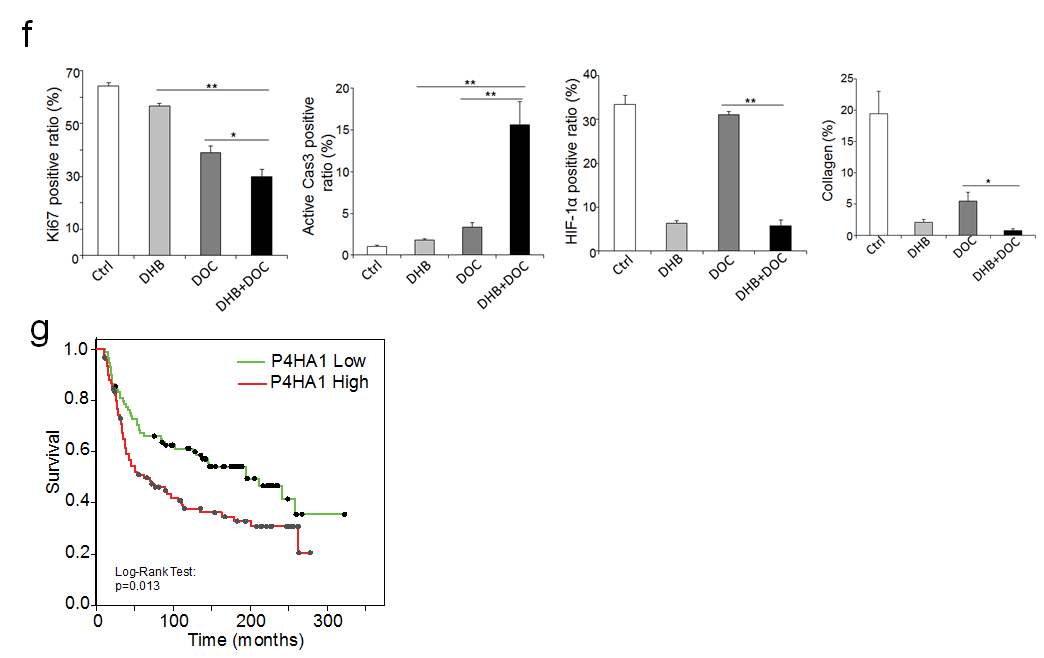
**

**Supplementary Figure 8. Inhibition of P4HA1 sensitizes TNBC to chemotherapeutic agent in TNBC PDO and PDX model. a, b** Growth of patient-derived tumor organoids (PDOs) in the 3D laminin-rich ECM gel was assessed after docetaxel or doxorubicin treatment in the presence of absence of P4HA inhibitor (DHB). **c** HE images, P4HA1 IHC staining images of tumor sections from TNBC patient and F1 mouse. Bar: 40 µm. **d** Image of four groups [group 1: control; group 2: DHB; group 3: docetaxel; group 4: DHB and docetaxel] tumors in TNBC PDX model. **e** HE images, Masson’s trichrome staining images, and IHC staining images (Ki67, active-Caspase 3, HIF-1α) of tumor sections from PDX model mice treated with DHB and/or docetaxel. Bar: 80 µm and 40 µm in enlarged images. **f** Quantification of cell proliferation, apoptosis, HIF-1α, and collagen deposition in tumor sections from mice treated with DHB and/or docetaxel. Results are presented as mean ± SEM. n=4. *, p < 0.05; **, p < 0.01, one-way ANOVA test. **g** P4HA1 expression is associated with poor prognosis in ER negative breast cancer after chemotherapy (METABRIC). The association of P4HA1 expression with overall survival was assessed by analyzing the mRNA levels in ER negative breast cancer tissues from patients who received chemotherapy, n=263, Kaplan-Meier survival analysis.


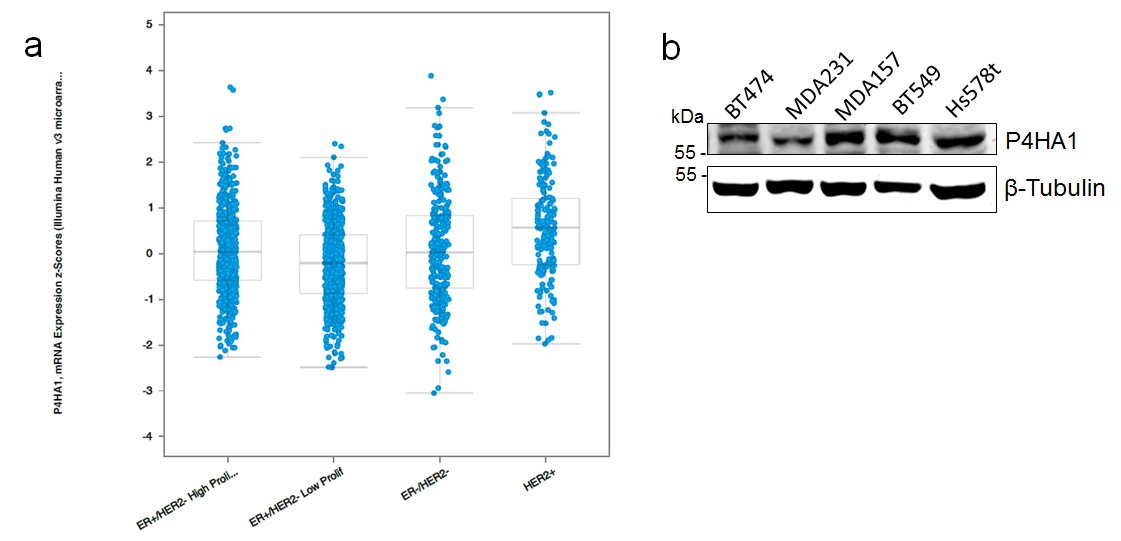


**Supplementary Figure 9**. **a** P4HA1 mRNA levels in three-gene classified subtypes; n=2509.  **b** P4HA1 protein levels in HER2 positive breast cancer cell line BT474 and TNBC cell lines.


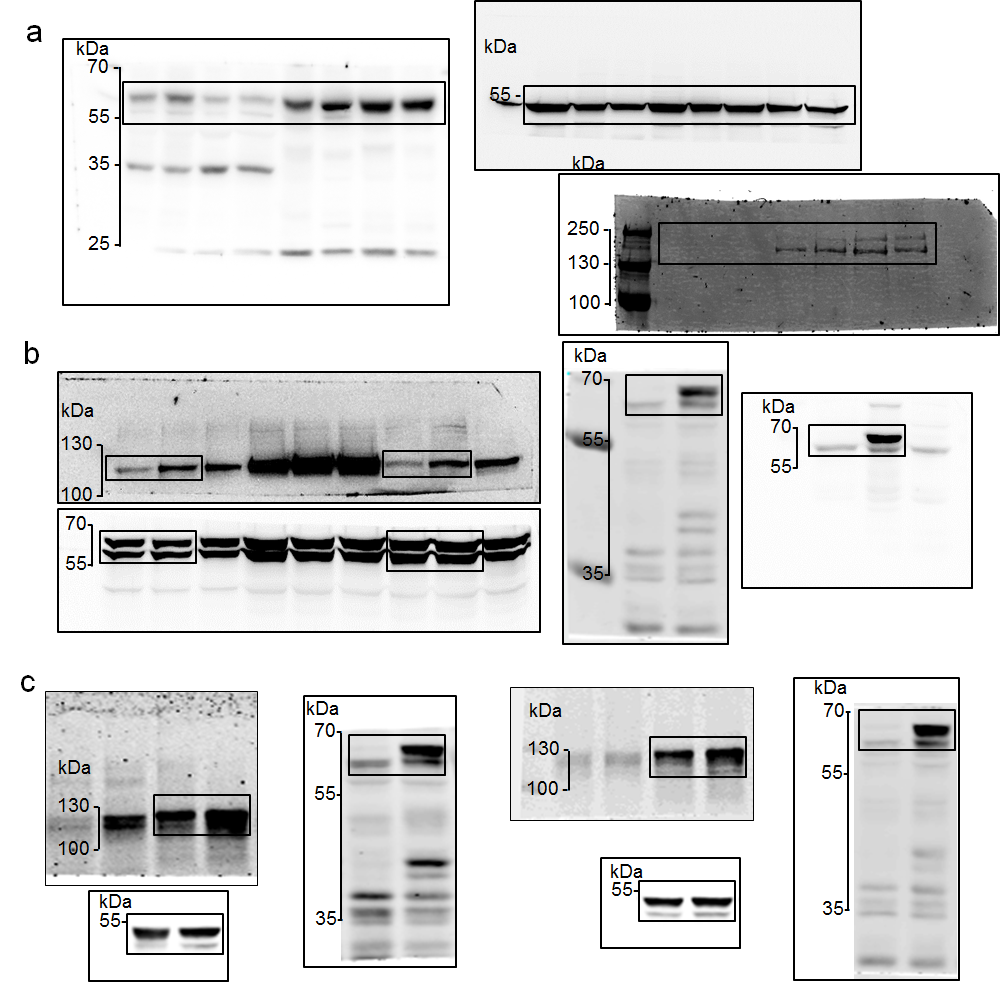


**Supplementary Figure 10. Uncropped blots corresponding to the main figures.** **a** Uncropped immunoblots for Figure 1d in the main text. **b** Uncropped immunoblots for Figure 2b in the main text. **c** Uncropped immunoblots for Figure 2c in the main text.

**
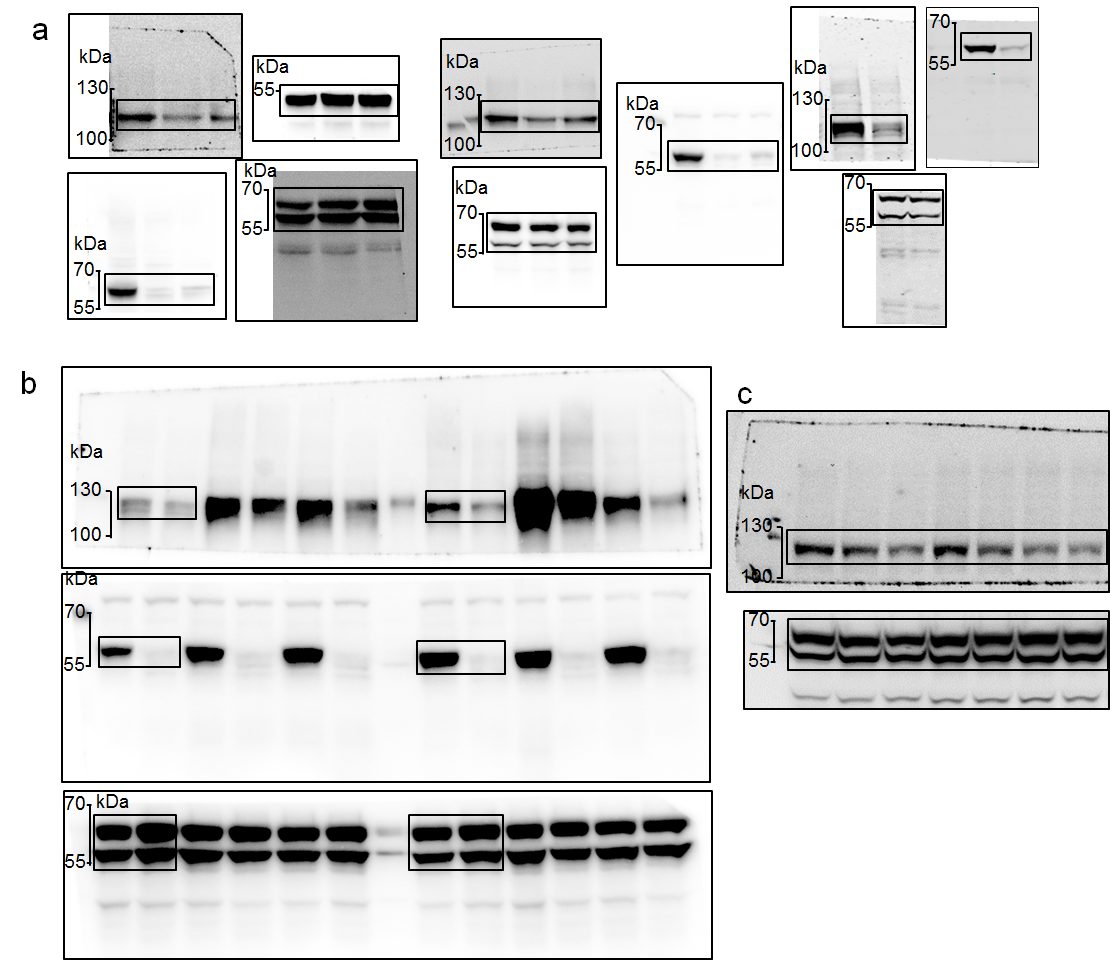
**

**Supplementary Figure 11. Uncropped blots corresponding to the main figures.** **a** Uncropped immunoblots for Figure 2e in the main text. **b** Uncropped immunoblots for Figure 2f in the main text. **c** Uncropped immunoblots for Figure 2g in the main text.

**
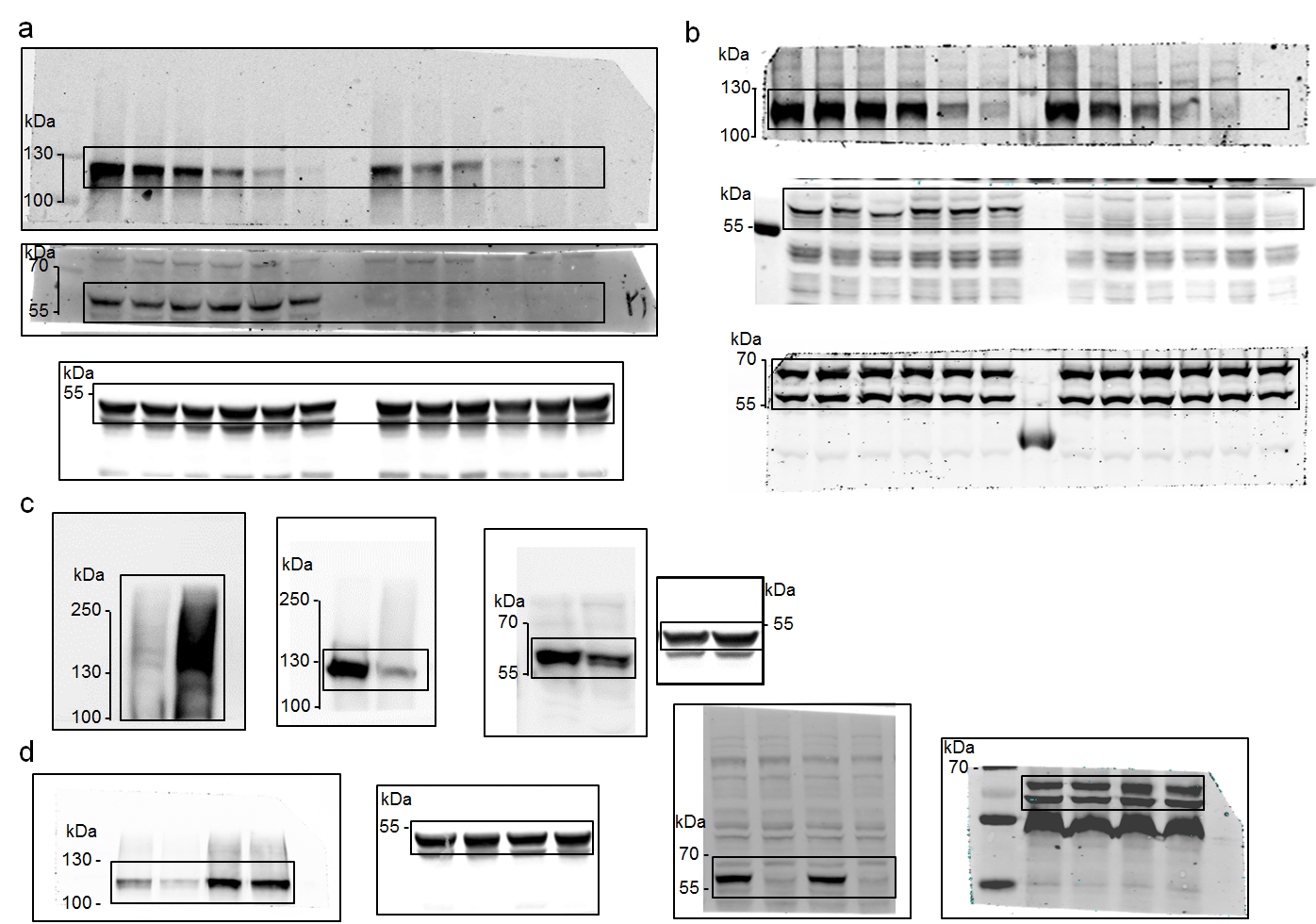
**

**Supplementary Figure 12. Uncropped blots corresponding to the main figures.** **a** Uncropped immunoblots for Figure 3a in the main text. **b** Uncropped immunoblots for Figure 3c in the main text. **c** Uncropped immunoblots for Figure 3e in the main text. **d** Uncropped immunoblots for Figure 3f in the main text.

**
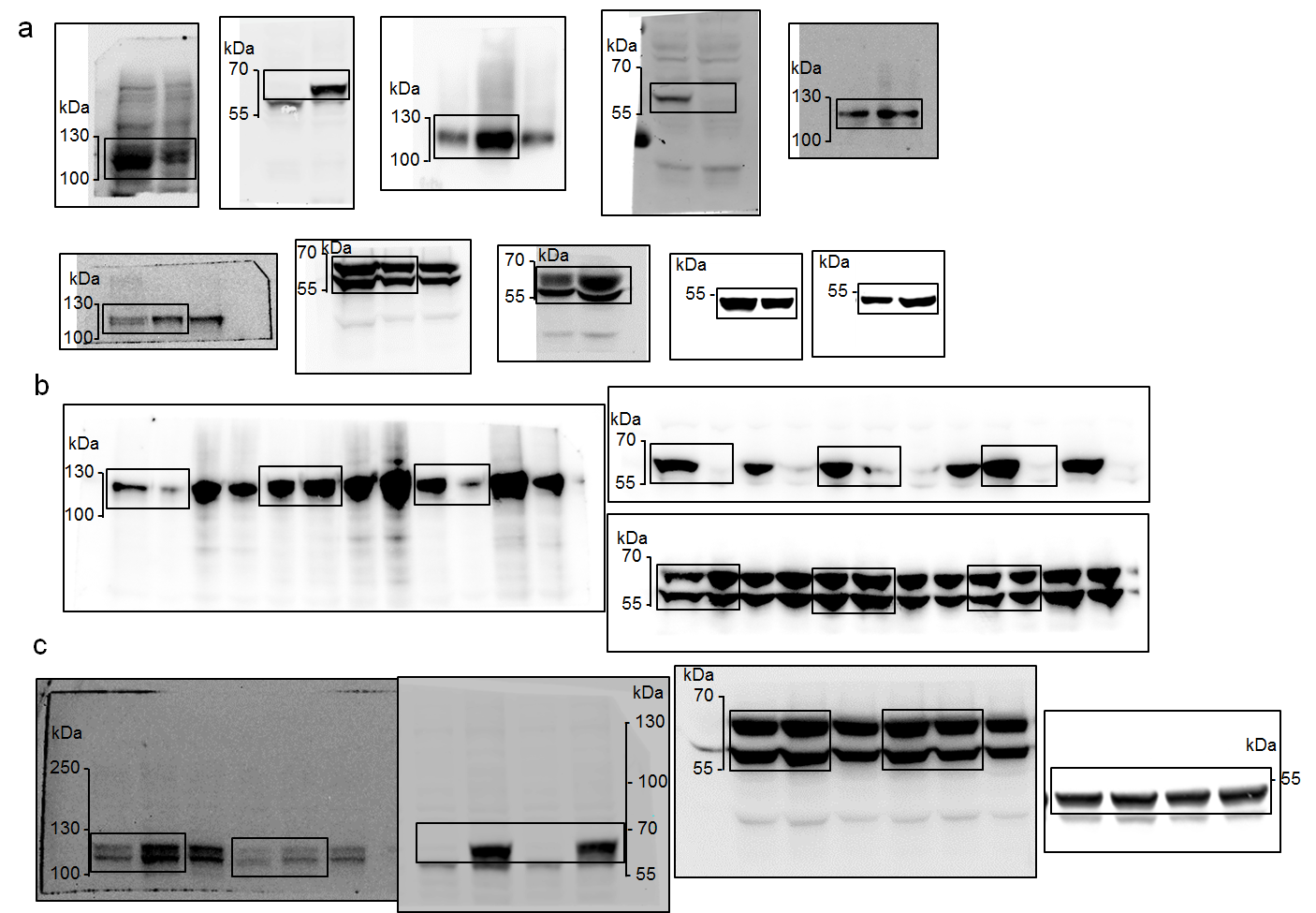
**

**Supplementary Figure 13. Uncropped blots corresponding to the main figures.** **a** Uncropped immunoblots for Figure 3g in the main text. **b** Uncropped immunoblots for Figure 3j in the main text. **c** Uncropped immunoblots for Figure 4f in the main text.


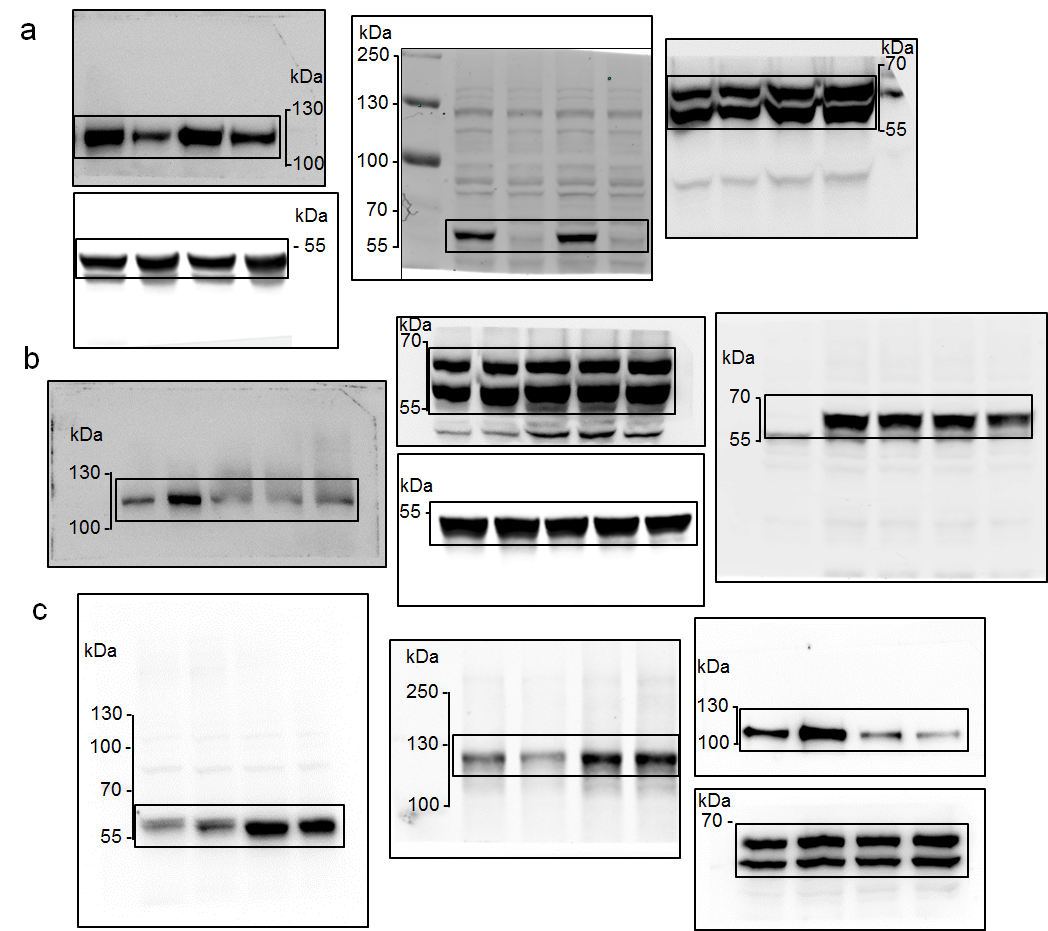


**Supplementary Figure 14. Uncropped blots corresponding to the main figures.** **a** Uncropped immunoblots for Figure 4g in the main text. **b** Uncropped immunoblots for Figure 4j in the main text. **c** Uncropped immunoblots for Figure 5a in the main text.

**
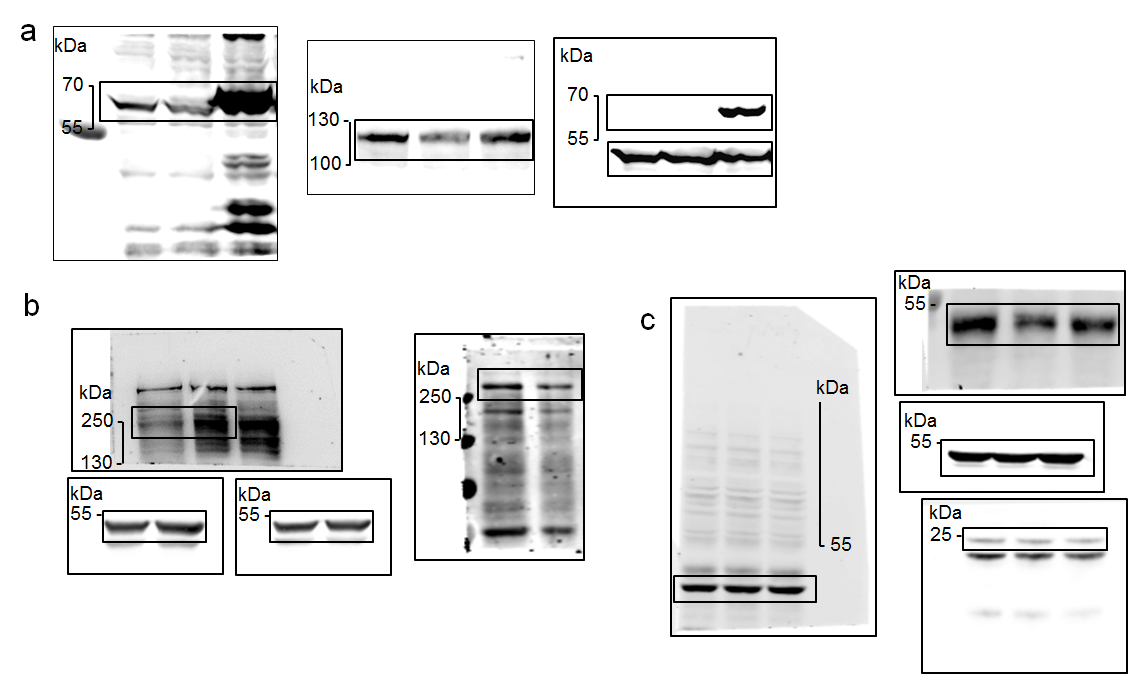
**

**
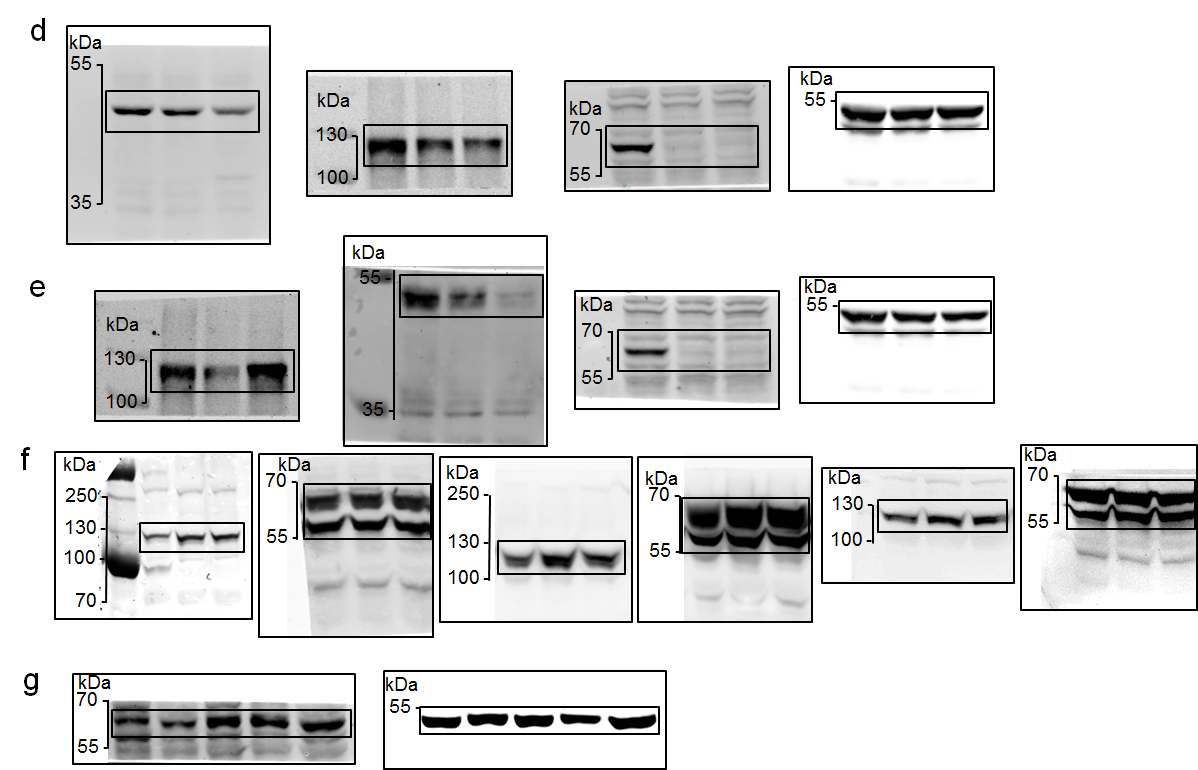
**

**Supplementary Figure 15. Uncropped blots corresponding to the Supplementary Figures.** **a** Uncropped immunoblots for Supplementary Figure 2b. **b** Uncropped immunoblots for Supplementary Figure 2c. **c** Uncropped immunoblots for Supplementary Figure 3a. **d** Uncropped immunoblots for Supplementary Figure 3b. **e** Uncropped immunoblots for Supplementary Figure 3c. **f** Uncropped immunoblots for Supplementary Figure 6b. **g** Uncropped immunoblots for Supplementary Figure 9b.

**Supplementary Tables**

**Supplementary Table 1**. Gene ontology analysis showed that P4HA1 expression was positively correlated with expression of “response to hypoxia” and “glycolysis” gene signatures. The P4HA1 co-expression genes were identified from the TCGA breast cancer dataset with cBioPortal (http://www.cbioportal.org/). Gene ontology analysis was performed with David (<https://david-d.ncifcrf.gov/home.jsp>) using the P4HA1 positive associated genes.

| **GO term** | **Gene count** | **p-value** | **Benjamini** |
| --- | --- | --- | --- |
| Response to Hypoxia | 10 | 2.80E-07 | 3.00E-04 |
| Glycolysis | 5 | 2.70E-04 | 9.60E-02 |
| Glucose metabolic process | 7 | 5.70E-04 | 1.50E-01 |

**Supplementary Table 2**. Reagents.

| **Reagents** | **Company** | **Catalog Number** |
| --- | --- | --- |
| 1,4-DPCA | Cayman | 331830-20-7 |
| 3,4-dihydroxybenzoic acid (DHB) | Sigma-Aldrich | 37580-25G-F |
| Docetaxel | TSZCHEM | RS019 |
| Doxorubicin | Cayman | 25316-40-9 |
| cycloheximide | Abcam | ab120093 |
| Bortezomib | Selleck | S1013 |
| octyl-α-ketoglutarate | Cayman | 876150-14-0 |
| dimethyl-succinate | Sigma-Aldrich | 398055-500G |
| Cisplatin | Selleckchem | S1166 |

#

#

# Supplementary Table 3. Primers.

| Gene | Primer name | Sequences (5’-3’) |
| --- | --- | --- |
| P4HA1 | Nhe1-F | AACGCTAGCGCCACCATGATCTGGTATATATTAAT |
|  | BamH1-R | ACTGGGATCCTTCCAATTCTGACAACGTAC |
|  | shP4HA-res-F | TATGAAAATCCTGTGGTCAGCCGAATTAATATGAGAATACA |
|  | shP4HA-res-R | TGTATTCTCATATTAATTCGGCTGACCACAGGATTTTCATA |
|  | H412S-F | GGACAGTATGAACCCTCTTTTGACTTTGCACGG |
|  | H412S-R | CCGTGCAAAGTCAAAAGAGGGTTCATACTGTCC |
|  | H483S-F | GATTATAGTACACGGTCTGCAGCCTGTCCAGTG |
|  | H483S-R | CACTGGACAGGCTGCAGACCGTGTACTATAATC |
|  | H501S-F | TCCAATAAATGGCTCTCTGAACGTGGACAAGAA |
|  | H501S-R | TTCTTGTCCACGTTCAGAGAGCCATTTATTGGA |
| P4HB | BamH1-F | ACTGGGATCCATGCTGCGCCGCGCTCTGCT |
|  | EcoR1-R | AATTGAATTCTTACAGTTCATCTTTCACAG |
| PDK1 | RTPCR-F | CTGTGATACGGATCAGAAACCG |
|  | RTPCR-R | TCCACCAAACAATAAAGAGTGCT |
| LDHA | RTPCR-F | AACATGGCAGCCTT TTCCTT |
|  | RTPCR-R | TTGCAGTTCGGGCTGTATTT |
| 18S rRNA | RTPCR-F | ACCTGGTTGATCCTGCCAGT |
|  | RTPCR-R | CTGACCGGGTTGGTTTTGAT |

**Supplementary Table 4**. Primary antibodies.

| **Antibody** | **Company** | **Catalog Number** | **Dilution** | **Species** |
| --- | --- | --- | --- | --- |
| Anti-HIF-1α | BD Biosciences | 610959 | WB: 1:1000  IHC: 1:100 | Mouse |
| Anti-Hydroxy-HIF-1α | Cell Signaling Technology | 3434P | WB: 1:1000 | Rabbit |
| Anti-Collagen I | Abcam | ab34710 | WB: 1:1000 | Rabbit |
| Anti-P4HA1 | Proteintech | 12658-1-AP | WB: 1:1000  IHC: 1:200 | Rabbit |
| Anti-HA | Sigma-Aldrich | H3663 | WB: 1:1000 | Mouse |
| Anti-Flag | Sigma-Aldrich | F1804 | WB: 1:1000 | Mouse |
| Anti-LaminA/C | Santa Cruz | sc-6215 | WB: 1:500 | Goat |
| Anti-β-Tubulin | Millipore Sigma | 05-661 | WB: 1:5000 | Mouse |
| Anti-PHD1 | Novus Biologicals | NB100-310SS | WB: 1:1000 | Rabbit |
| Anti-PHD2 | Cell Signaling Technology | 4835S | WB: 1:1000 | Rabbit |
| Anti-PHD3 | Novus Biologicals | NB100-139SS | WB: 1:1000 | Rabbit |
| Anti-P-Stat3 | Cell Signaling Technology | 9135 | WB: 1:1000 | Rabbit |
| Anti-Stat3 | Cell Signaling Technology | 4904S | WB: 1:2000 | Rabbit |
| Anti-CD44 | Invitrogen | PIMA513890 | WB: 1:1000 | mouse |
| Anti-CD24 | Invitrogen | PIMA511833 | WB: 1:1000 | mouse |
| Anti-Ecad | BD Biosciences | 610181 | WB 1:5000 | Mouse |
| Anti-KI67 | Abcam | ab15580 | IHC: 1:200 | Rabbit |
| Anti-Active Caspase3 | Millipore | AB3623 | IHC: 1:100 | Rabbit |
| Anti-ER | Dako | IR151 | ready to use | Rabbit |
| Anti-PR | Dako | IR068 | ready to use | Mouse |
| Anti-HER2 | Dako | A0485 | IHC: 1:100 | Rabbit |

**Supplementary Methods**

**Cell Culture, virus preparation and reagents used in the study**

# Human breast cancer cell line MDA-MB-231 cells (American Type Culture Collection) were maintained in Dulbecco’s Modified Eagle’s Medium (DMEM)/F12 (Sigma Aldrich) with 10% fetal bovine serum (Sigma Aldrich), 10 units/ml of penicillin and 0.1 mg/ml of streptomycin (Invitrogen). Human breast cancer cell line Hs578T cells (American Type Culture Collection), T47D cells (American Type Culture Collection), ZR75 cells (American Type Culture Collection), MDA157 cells (American Type Culture Collection) and BT474 cells (A kind gift from Dr. Qingbai She) were maintained in Dulbecco’s Modified Eagle’s Medium (DMEM) (Sigma Aldrich) with 10% fetal bovine serum (Sigma Aldrich), 10 units/ml of penicillin and 0.1 mg/ml of streptomycin (Invitrogen). BT549 cells (American Type Culture Collection) were maintained in RPMI 1640 Medium (Sigma Aldrich) with 10% fetal bovine serum (Sigma Aldrich), 10 units/ml of penicillin and 0.1 mg/ml of streptomycin (Invitrogen). HEK293 FT cells (A kind gift from Dr. Mina J Bissell (Lawrence Berkeley Natl Laboratory)) were maintained in Dulbecco’s Modified Eagle’s Medium (DMEM) (Sigma Aldrich) with 10% fetal bovine serum (Sigma Aldrich), 0.1 mM Non-Essential Amino Acids (Hyclone), 6 mM L-glutamine (Sigma Aldrich), 1 mM Sodium Pyruvate (Gibco), 10 units/ml of penicillin and 0.1 mg/ml of streptomycin (Invitrogen). MCF10A cells (A kind gift from Dr. Michael W Kilgore) were maintained in Dulbecco's Modified Eagle's Medium (DMEM)/F12 (Sigma Aldrich) with 5% horse serum, 20ng/ml EGF, 0.5 mg/ml Hydrocortisone, 100 ng/ml Cholera Toxin, 10μg/ml Insulin, 10 units/ml of penicillin and 0.1 mg/ml of streptomycin (Invitrogen). HMLE cells (American Type Culture Collection) were maintained in MEGM™ Mammary Epithelial Cell Growth Medium (Lonza, CC3150). All cells were treated with Plasmocin™ (Invivo Gen) to eliminate and prevent mycoplasma contamination. For normal culture condition, cells were cultured at 5% CO2, 95% O2 at 37°C. For hypoxia condition, cells were cultured at 5% CO2, 2% O2, within an O2-controlled chamber (Modular Incubator Chambers, Billups-Rothenberg, Inc).

# Flag-tagged HIF-1α wide-type (WT) cDNA and HIF-1α mutant (P402A, P564A) cDNA were sub-cloned from pBabe-HIF-1α-WT-HA and pBabe-HIF-1α-P402A, P564A-HA (from Dr. Tianyan Gao) into pCDH1 plasmid and generated expression vector pCDH1-HIF-1α(WT)-Flag and pCDH1-HIF-1α(MT)-Flag. P4HA1 and P4HB cDNA clones were purchased from Thermo Fisher Scientific. P4HA1 cDNA was cloned into pCDH1 plasmid and generated expression vector pCDH1-P4HΑl-Flag. P4HB cDNA was cloned into pBabe plasmid and generated vector pBabe-P4HB-neo. P4HA1 Knockdown plasmids shP4HA1were purchased from Sigma (Clone ID: NM_000917.3-1415s21c1 and NM_000917.3-870s21c1). To rescue P4HA1 expression P4HA1-silenced cells, shP4HA1A-resistant P4HA1 cDNA was cloned into pCDH1 plasmid. Primers information is listed in Supplementary Table 2. HEK293 FT cells were transfected with pCDH1, pBabe or shRNA vector (Sigma) plus lentivirus packaging vectors using lipofectamine (Invitrogen). Culture supernatants containing viral particles were collected 48 h after transfection. Cancer cells were infected with lentivirus and selected by puromycin or G418 48h after infection.

**Tissue microarray and Immunohistochemistry analysis**

Protein levels of P4HA1 expression in human breast cancer tissues were determined using tissue microarray (TMA). Each case in the TMA is represented by three separate tissue cores. IHC scoring results from each of these three tissue cores were averaged to produce a final score for each case. P4HA1 immunohistochemical staining was scored according to a standard semi-quantitative scale as follows: negative (0), weak (1), moderate (2), strong (3). Scoring was performed by a pathologist who was blinded to clinical and pathologic variables.

Xenograft tumor sections were deparaffinized and rehydrated through 100% alcohol, 95% alcohol, 70% alcohol to PBS solution. Endogenous peroxidase was blocked by incubation with 3% H_2_O_2_ for 20 min. At the antigen retrieval step, slides were steamed in citrate sodium buffer for 30 min. Slides were blocking with Avidin/Biotin Blocking Kit (Vector Laboratories, SP-2001) incubated with primary antibodies (Anti-HIF-1α, BD Biosciences, 610959, 1:100; Ki67, Spring Bioscience, M3060, 1:500; Active-caspase3, Millipore, 1:100; Anti-ALDH1A1, 1:100) at 4°C overnight, and then the sections were incubated with Biotinylated Goat Anti-Mouse IgG Antibody (Vector Laboratories, BA-9200) or Biotinylated Goat Anti-Rabbit IgG Antibody (Vector Laboratories, BA-1000) at room temperature for 60 min. After incubated with Streptavidin, Horseradish Peroxidase (Vector Laboratories, SA-5704) at room temperature for 60 min, slides were added substrate diaminobenzidine (DAB, Vector Laboratories, SK-4100), images were taken by Nikon Eclipse 80i microscope.

**Masson's Trichrome Staining**

Xenograft tumor sections were deparaffinized and rehydrated through 100% alcohol, 95% alcohol, 70% alcohol to water. Samples were re-fixed in Bouin's solution at 60 °C for 60 min, stained in Weigert's working hematoxyin for 10 min, and then stained in Biebrich scarlet-acid fuchsin solution for 5 min. Sections were incubated in phosphomolybdic/phosphotungstic acid solution for 10 min, and then were transferred to aniline blue solution and incubated for 5 min. Then sections were incubated with 1% acetic acid for 1 min and washed in distilled water. Images were taken with a Nikon Eclipse 80i microscope.
